# Recent Trends in Synthesis of Chloramphenicol New Derivatives

**DOI:** 10.3390/antibiotics10040370

**Published:** 2021-03-31

**Authors:** Anna N. Tevyashova

**Affiliations:** Gause Institute of New Antibiotics, 11 B. Pirogovskaya, 119021 Moscow, Russia; chulis@mail.ru; Tel.: +7-499-246-06-36

**Keywords:** chloramphenicol, synthesis, antibacterial activity, peptides, antibiotic resistance, side effects, translation, ribosome, antiproliferative activity, antimicrobial peptides

## Abstract

Chloramphenicol (CAM), the bacteriostatic broad-spectrum antibiotic, isolated from *Streptomyces venezuelae* during the “golden era” of antibiotic discovery, nowadays has limited clinical potential due to adverse side effects and frequent antimicrobial resistance. Numerous CAM analogs were synthesized in order to find the derivatives with improved pharmacological properties and activity on resistant bacterial strains. This work aims to summarize the most recent achievements in obtaining new CAM analogs reported during the last five years. Current investigations are mainly focused on elucidating the molecular basis of the mode of CAM action and determining the mechanisms of resistance to this class of antibiotics or on studies of the possible use of the CAM scaffold to search for therapeutic agents with different CAM modes of action—such as selective antiproliferative agents or bacterial cell wall biosynthesis inhibitors. Hopefully, a deeper understanding of the CAM interactions with the target and its specificity will generate research ideas for developing new effective drugs.

## 1. Introduction

**Figure 1:**
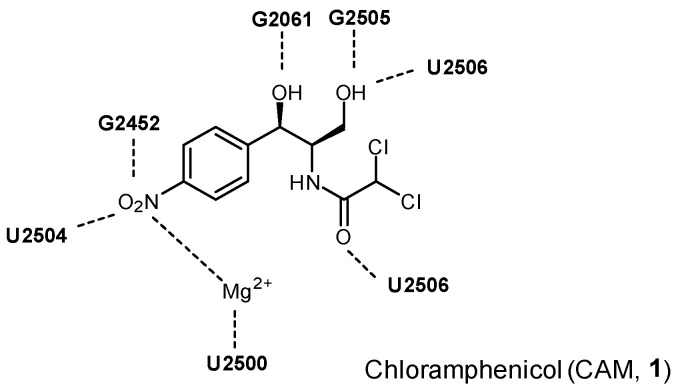
Structure of chloramphenicol and its interactions with the 23S rRNA nucleotides.

Chloramphenicol (CAM) is a bacteriostatic broad-spectrum antibiotic that was isolated from *Streptomyces venezuelae* in 1947 during the “golden era” of antibiotic discovery [1]. CAM consists of a *p*-nitrobenzene ring connected to a dichloroacetyl tail through a 2-amino-1,3-propanediol moiety; only the D-threo isomer isolated from natural sources demonstrates antimicrobial activity (Figure 1) [2]. In fact, CAM is actually among the few natural compounds that have two strong electron-withdrawing groups—nitro and halogen.

CAM is a competitive translation inhibitor as its binding with the peptidyltransferase center (PTC) in the 23S rRNA of the 50S subunit of the bacterial ribosome prevents the interaction of an incoming aminoacyl moiety of an aminoacyl-tRNA substrate with the A site [3]. Both hydroxy groups of the antibiotic, as well as the carbonyl group of the dichloroacetamide residue, form a hydrogen bond with the 23S rRNA nucleotides (Figure 1) which results in the inhibition of the peptide bond formation [4].

A variety of mechanisms of resistance to CAM has been determined; however, the main limitation of the clinical potential of CAM is related to its side effects which include neurotoxicity and hematologic disorders [5]. The essential need for new antimicrobial agents caused by the rise of multidrug-resistant bacteria has attracted attention to the “old” classes of antibiotics and prompted efforts to find new, effective, and less toxic CAM derivatives. A relatively recent review, published in mid-2016, exhaustively covered efforts to synthesize CAM derivatives with improved pharmacological properties [6]. Therefore, the current work is an effort to summarize new CAM analogs discovered or obtained since this work was published.

## 2. New CAM Derivatives

### 2.1. O-acyl Derivatives of Chloramphenicol

**Figure 2:**
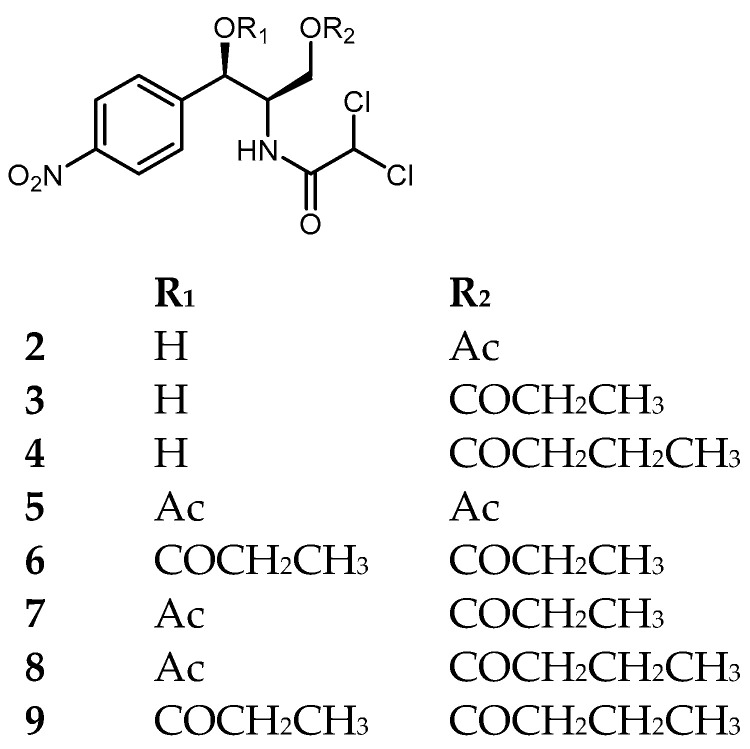
Structures of chloramphenicol (CAM) derivatives discovered by functional metagenomics.

Screening of a soil library revealed seven metagenomic clones that were capable of modifying CAM to mono- or diacylated CAM derivatives **2**–**9** and demonstrated antibacterial activity against methicillin-resistant *Staphylococcus aureus* (MRSA) (Figure 2) [7]. Three of the obtained compounds, 1′-acetyl-3-propanoylchloramphenicol (**7**), 1′-acetyl-3′-butanoylchloramphenicol (**8**), and 3′-butanoyl-1′-propanoylchloramphenicol (**9**) have not been described previously.

Supposedly, CAM undergoes enzymatic acylation by acyltransferases in the clones yielding compounds **2**–**9**. Isolated compounds **2**–**7** did not possess antibacterial activity (minimal inhibitory concentrations (MIC) > 200 μg/mL) against MRSA ATCC 1708, *Pseudomonas aeruginosa*, and *Escherichia coli*, though they demonstrated moderate activity towards the MRSA EAMC30 strain used for initial screening.

**Scheme 1:**
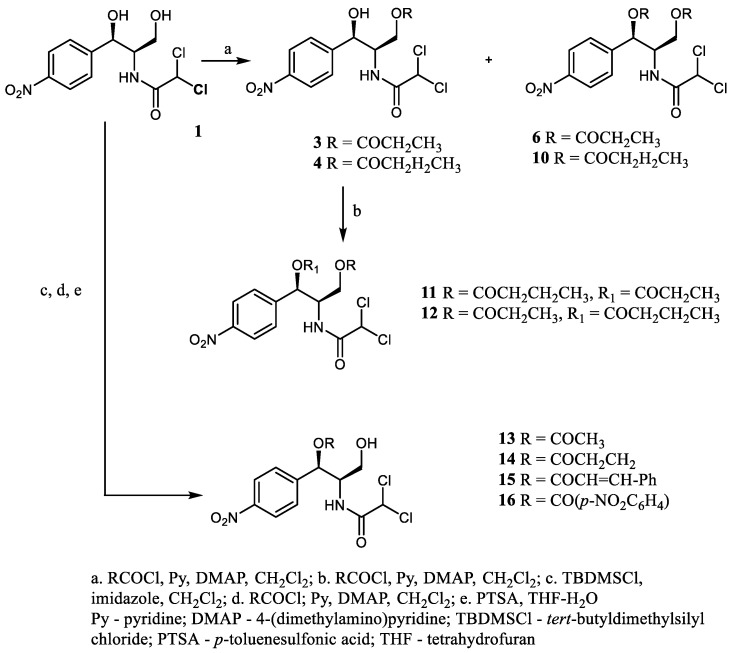
Synthesis of the 1-*O*-acyl and 1,3-*O*-diacyl derivatives of CAM.

Among a series of natural (**2**–**7**) or synthetically obtained (**10**–**16**) 1-*O*-acyl and 1,3-*O*-diacyl CAM derivatives (Scheme 1), 1′-(*p*-nitrobenzoyl)chloramphenicol (**16**) demonstrated the highest activity towards *Mycobacterium intracellulare* and *Mycobacterium tuberculosis* with MICs values of 12.5 and 50.0 μg/mL versus 25 and >100 μg/mL for CAM (**1**), respectively (Table 1). The equal potency of **1** and mono acetyl compounds **2** and **13** against *M. intracellulare* (MICs = 25.0 μg/mL) indicated that the monoacylation of either the 1- or 3-hydroxy group of the antibiotic did not alter its antimycobacterial activity against the tested strain.

The hypothesis of enzymatic acylation of CAM (**1**) was supported by the fact that the expression of metagenomics-encoded esterases or a cloned gene that induced BAC vector copy number allowed for novel *O*-acyl-CAM derivatives from the exogenously supplied CAM (**1**) to be obtained.

**Figure 3:**
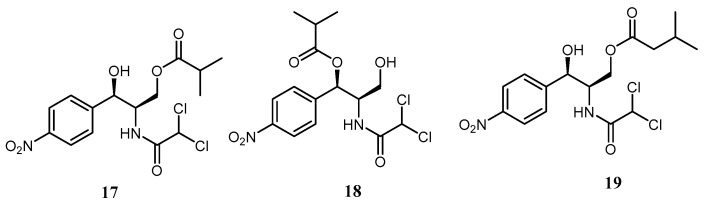
*O*-Acyl CAM derivatives identified from *L. enzymogenes*.

The acetylation of the primary hydroxyl group of CAM is a well-established mechanism of drug resistance in *E. coli* and *Streptomyces* spp. [8], but recently it was found that *Lysobacter enzymogenes*, a biocontrol agent with intrinsic resistance to multiple antibiotics, contains a pool of unusual acyl donors for enzymatic modification of CAM [9]. 3’-Isobutyroylchloramphenicol (**17**), a new compound 1’-isobutyroylchloramphenicol (**18**), and 3’-isovaleroylchloramphenicol (**19**) (Figure 3) were identified in a chloramphenicol acetyltransferase gene containing mutant of *L. enzymogenes* as well as in the wild type.

Presumably, the available acyl-CoA cellular pool in *L. enzymogenes* varies from that in other bacteria which resulted in a difference in the identified CAM acylation products. Moreover, it has been established that the global regulator *clp* gene and the Gcn5-related N-acetyltransferase (GNAT) are responsible for the observed resistance of *L. enzymogenes* to CAM (**1**).

**Scheme 2:**
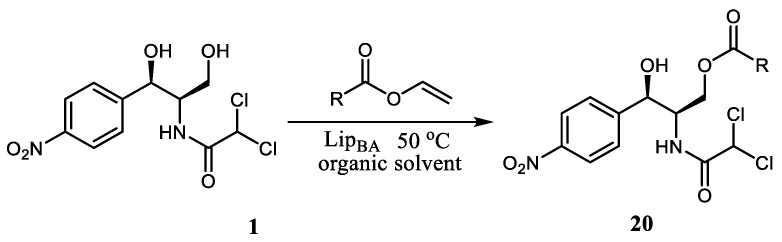
Selective acylation of the primary hydroxyl group of CAM with the lipase from *B. amyloliquefaciens*.

Other research has demonstrated that Lip_BA_ (lipase cloned from *Bacillus amyloliquefaciens*) can be useful for the synthesis of chloramphenicol esters (**20**) with different carbon chain lengths (Scheme 2) [10]. The vinyl propionate proved to be the best among the acyl donors with different carbon chain lengths, as it allowed CAM ester with the highest yield, conversion (98%), and purity (99%) to be obtained under the same reaction conditions. The optimized conditions included the use of 1,4-dioxane as a solvent, 5:1 molar excess of the vinyl propionate, and 4 g/L concentration of Lip_BA_. The reaction was carried out for 8 h at 50 °C.

Thus, the suggested approach, involving enzymatic acylation of CAM, allows selective modification of the hydroxyl group of the antibiotic without the protection of other functionalities. It also demonstrated the perspective of the lipase Lip_BA_ as a catalyst for modification of complex molecules in accordance with the principles of green chemistry.

**Scheme 3:**
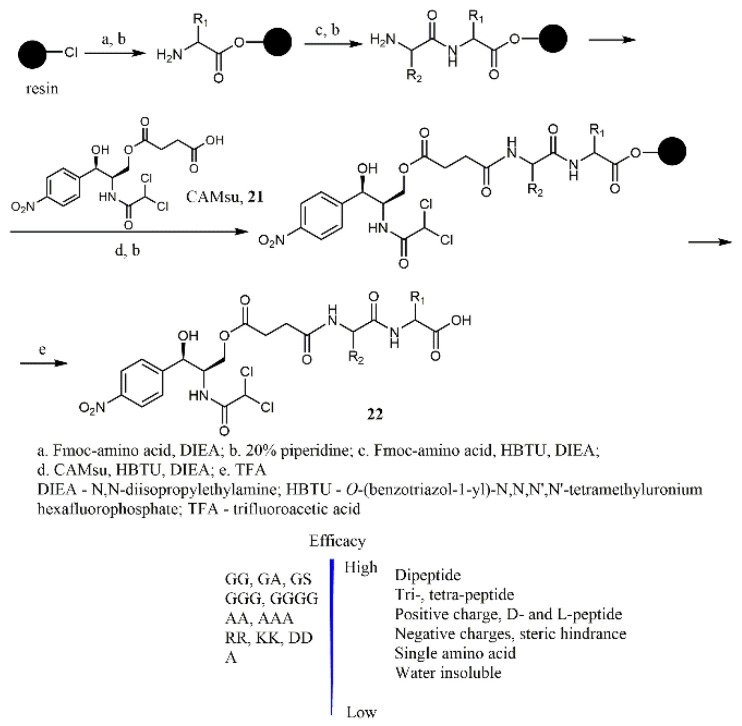
Synthesis of the peptide-conjugated chloramphenicol analogs.

It is established that ester derivatives of CAM on the primary hydroxyl group are unstable in vivo, and *O*-acyl derivatives are rapidly converted to the parent drug [11]. Chloramphenicol succinate (CAMSu, **21**), a clinically approved drug, acts as a prodrug as it is hydrolyzed to the active CAM while circulating in the body. Wang et al. described 34 structural analogs of chloramphenicol (**22**) in which different peptides were conjugated to the CAM molecule via the succinate spacer (Scheme 3) and examined their activities against *E. coli* [12].

At the first stage, the fluorenylmethyloxycarbonyl (Fmoc)-protected amino acid was attached to the 2-chlorotrityl chloride resin, then the Fmoc group was removed and the resulting conjugates interacted either with the next amino acid or CAMsu. Cleavage of the target conjugate from the resin was performed using trifluoroacetic acid (TFA). Different types of CAMsu-peptide conjugates have been obtained, including a series of derivatives with neutral peptides of different lengths (**22a**–**22q**), charged peptides (**22aa**–**22aj**), and naphtyl-containing peptides (**22ba**–**22bc**) (Table 2). Moreover, CAMsu conjugates containing a single amino acid (**22ca**), OMe-cap (**22cb**), a sulfate (**22cc**), or a phosphate group (**22cd**) have been synthesized. Research has been established that conjugating peptides to CAMsu effectively modulates the intracellular hydrolysis of the ester bonds, regenerating the antibiotic against *E. coli* (Table 2). The lower hydrolysis rate of the ester bond in the conjugates, whose side chains of peptides were bearing negative charges or were sterically hindered, correlated with their lower antibacterial activities in comparison with the parent CAM (**1**). Dipeptide conjugates most effectively increased the efficacy of CAMsu in a row from single amino acid to pentapeptide substituents. The presence of the D-amino acid residues slightly increased the hydrolysis rate of the ester bond in the prodrugs in comparison with the corresponding L-amino acids analogs.

Conjugates of chloramphenicol succinate with comparatively hydrophilic neutral peptides (such as Gl, Ga, GS, etc.) demonstrated the highest inhibitory activities (MICs 20–200 μM) which were comparable with MICs values for CAM (**1**). However, the obtained peptides-CAMsu conjugates, like the prodrug CAMsu, generally demonstrated lower cytotoxicity than CAM (**1**) against the HS-5 cell line while their cytotoxicity against HEK292 cells was almost the same as for CAM (**1**). Thus, conjugating CAM (**1**) with peptides can be an effective approach for modulating the properties of the CAM prodrugs.

### 2.2. Modification of the Amino Group of Des-Dichloroacetyl CAM (CAM Amine)

**Scheme 4:**
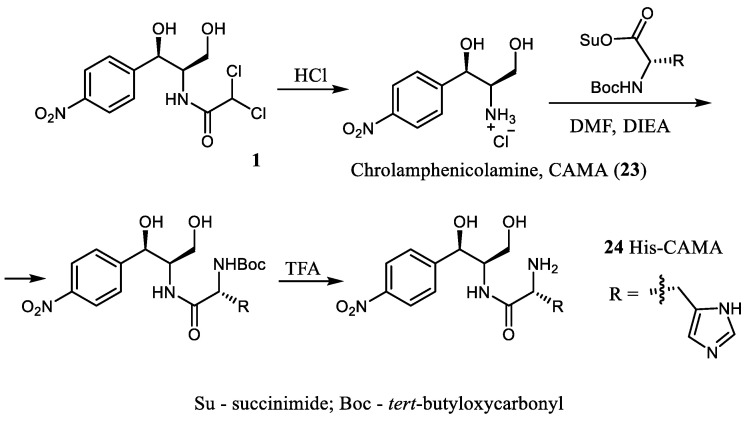
Synthesis of the amino acid chloramphenicol derivatives.

Aiming to explore new inhibitors of translation, a series of CAM analogs aminoacylated with different amino acids, including the N-protected ones, has been described [13]. The synthetic approach was based on an acylation of CAM amine (CAMA, **23**), an inactive CAM derivative, with activated amino acids (Scheme 4).

A competition-binding assay with the use of BODIPY-labeled erythromycin (BODIPY-ERY) was exploited to evaluate the affinity of the synthesized amino acid, CAM analogs, with the ribosome. Though most of the synthesized amino acid CAM derivatives were less potent translation inhibitors than the parent antibiotic CAM (**1**), the analog **24**, which contained the L-histidyl residue, demonstrated 10-fold higher affinity to the ribosome than CAM (apparent dissociation constants with the *E. coli* 70S ribosome K_Dapp_ were 0.24 ± 0.06 µM for compound **24** vs. 2.8 ± 0.5 for **1**). In general, the presence of a positive charge (i.e., unprotected α-amino group) and the small size of the amino acid side chain were preferable for binding to the ribosome. Nevertheless, the ability of the synthesized compounds to inhibit translation did not correlate with their affinity to the vacant ribosome.

**Figure 4:**
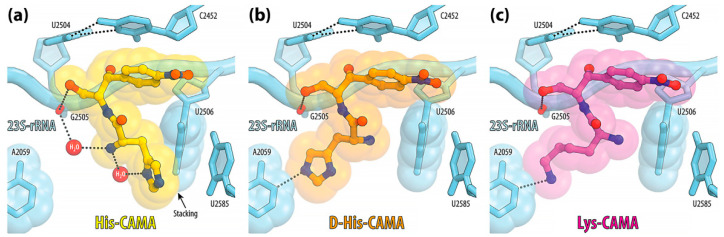
Side chain specific interaction of amino acid CAM analogs with the ribosome. Compound-specific H-bond interaction of His-CAMA (**24**, His-CAM) (**a**), D-His-CAMA (**b**), or Lys-CAMA (**c**) with the nucleotides of the 23S rRNA are indicated with dashed lines. Stacking interactions of 24 are shown with the black arrow (the picture is published with the permission of the Elsevier, Order Number: 5033250763121).

Studies of the complexes of L-His-CAMA (**24**), D-His-CAMA, or L-Lys-CAMA with *Thermus thermophilus* 70S ribosome by X-ray method revealed that the tested analogs specifically interacted with the nucleotides of the 23S rRNA at the PTC, wherein the position of the amphenicol residues of the conjugates was identical to the binding position of the parent antibiotic CAM (**1**), while the introduced aminoacyl moiety was oriented towards the upper part of the peptide exit tunnel (Figure 4).

The additional interactions observed in the case of compound **24**, i.e., π-stacking interactions of the histidine side chain with the U2506 and formation of the hydrogen bond between the α-amino group and the phosphate group of G2505, are possible explanations of the increased affinity of **24** to the ribosome in comparison with the parent CAM (**1**). The affinities of the D-His-CAMA and Lys-CAMA to the ribosome were lower in comparison with **24**, most probably due to the fact that no hydrogen bond between the α-amino group and the phosphate group of G2505 was observed.

Presumably, His-CAMA analog (**24**) could possess antibacterial activity to the strain resistant to CAM due to methylation of the A2503 of the 23S rRNA by Cfr-methyltransferase [14] or due to the mutation of A2503 to G [15] as in silico modeling demonstrated that placement of compound **24** in the PTC was somewhat shifted relative to the parent antibiotic CAM (**1**), which could help avoid the collision with the C8-methyl group of the A2503 residue in the modified Cfr-ribosome and also results in less susceptibility to the nucleotide mutation. However, no data on the antibacterial activity of the amino adic derivatives of CAM have been published yet.

**Scheme 5:**
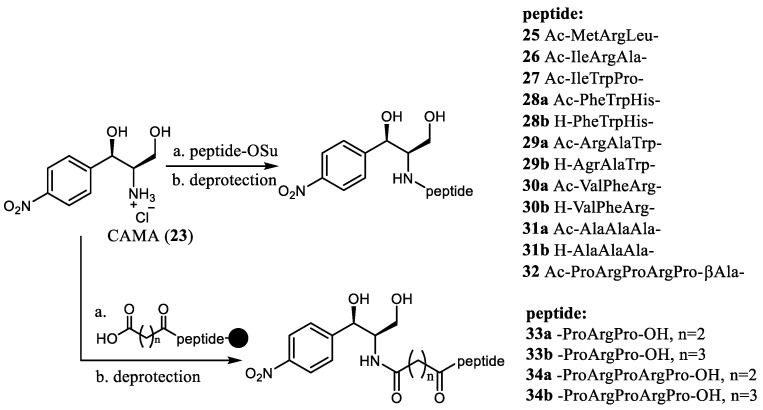
Synthesis of peptide chloramphenicol analogs.

A similar scheme was employed for the synthesis of the chloramphenicol amine peptide conjugates with regulatory “stop peptides” (i.e., MRL, IRA, IWP) (**25**–**27**) [16] or cationic peptides (**28**–**34**) [17] (Scheme 5). In general, this approach is based on the conjugation of the ribosomal antibiotics that contain amino acid and peptides which mimics the nascent polypeptide chain [18]. Studies of the interaction of such analogs with the ribosome are employed for establishing the specialties of recognition of the peptide chain by the ribosome tunnel in addition to the mechanisms of translation regulation. The starting compound, CAMA (**23**), was acylated by the OSu-activated N-acetyl peptides. Then, the deprotection of the functional groups in the side chains was carried out, resulting in novel semisynthetic CAM derivatives that contain the tripeptide, which models the 3′-terminal region of the peptidyl-tRNA (**25**–**27**), the antimicrobial peptide (i.e., oncocin, metalnikovin, bactenecin), or their synthetic analogs (**28**–**32**) (Scheme 5). In some cases, the N-Ac protecting group was also removed to obtained corresponding **28b**–**31b** derivatives with the unsubstituted N-terminus amino group. To obtain chloramphenicol amine derivatives containing sequences common to the proline-rich antimicrobial peptides in their structure (**33, 34**), a solid phase synthesis scheme was developed. The interaction of the obtained new CAM conjugates **25**–**34** with the ribosome was studied by the molecular docking-based modeling as well as by the displacement of the fluorescent erythromycin analog from its complex with *E. coli* ribosomes in order to find possible contacts of the ribosomal tunnel with the peptide residues. The affinity of compounds (**25**–**27**) to the *E. coli* ribosome was very close to that of the parent antibiotic CAM (**1**). The cationic tripeptides, especially RAW-CAMA (**29b**), demonstrated the highest ability to bind with the *E. coli* ribosome. Results of the chemical probing and in silico calculations indicated that the highest inhibitory activity of the new analog **29b** could be explained by its similarity to the parent CAM (**1**) mechanism of action. It was demonstrated that, like CAM (**1**), the peptide derivative RAW-CAMA (**29b**) forms π-interactions between the side chain of the arginine residue and the A2062 and between the tryptophan-residue and G2505, while no interaction with A2058 was determined.

The chloramphenicol analog **29b** demonstrated similar activity as the parent antibiotic CAM (**1**) in an in vitro protein biosynthesis inhibition assay. Although no data on antibacterial activity of compounds **25**–**34** were reported, substances of this type are quite promising for studying the interaction between regulatory or antimicrobial peptides and the ribosomal tunnel, either biochemically or structurally.

**Scheme 6:**
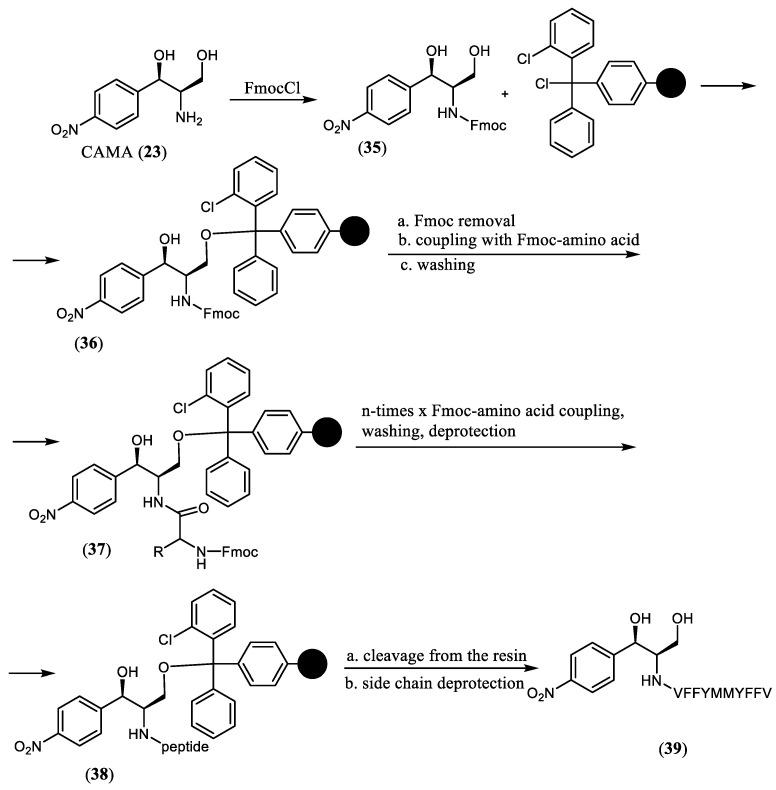
Synthesis of decapeptide chloramphenicol analog.

Bougas et al. synthesized and investigated a conjugate of CAM with decapeptide which demonstrated a dual action on the ribosome, being able to bind with both PTC and the polypeptide exit tunnel [19]. The study aimed to improve the previously described oligopeptide derivative of chloramphenicol amine, the MYFFV-CAMA, which demonstrated the ability to inhibit the ribosome activity in vitro by occupying the classical chloramphenicol binding site with the chloramphenicol residue and the peptidyl moiety directing towards the tunnel [18]. With the aim to set up an additional interaction of the peptide chain with the hydrophobic crevice of the ribosomal tunnel, the palindromic peptide sequence VFFYM-MYFFV was designed and constructed. A solid-phase strategy was employed to obtain the target derivative, VFFYM-MYFFV-CAMA (**39**) (Scheme 6). The amino group of the starting CAMA (**23**) was protected by the Fmoc-group, and the obtained compound **35** was bound to a 2-chlorotrityl-chloride resin. Then, the protected amino acids were added stepwise following the Fmoc-protocol. After conjugation with the last amino acid, the obtained conjugate was cleaved from the solid support and deprotected to give the target derivative **39**. The peptidyl transferase inhibitory activity of the newly synthesized CAM analog **39** determined on different translation systems was less than the corresponding activity of the parent CAM (**1**). Nevertheless, compound **39** demonstrated an inhibition activity on a model of green fluorescent protein synthesis in a coupled in vitro transcription-translation assay as well as an inhibitory action on lysine polymerization in a poly(A)-programmed ribosome that can possibly be explained by the existence of an additional target for compound **39** in comparison with **1**. Since obtained results supported the interaction of the new analog **39** with the A2058 and the A2059 located near the entrance of the tunnel, this finding can be the evidence of possible dual action of **39**, both on the peptidyl transferase center area and within the tunnel, both essential for ribosomal functions.

Thus, conjugation of CAM with oligopeptides offers new opportunities to modulate translation inhibition of specific proteins by varying the oligopeptide length and sequence.

**Scheme 7:**
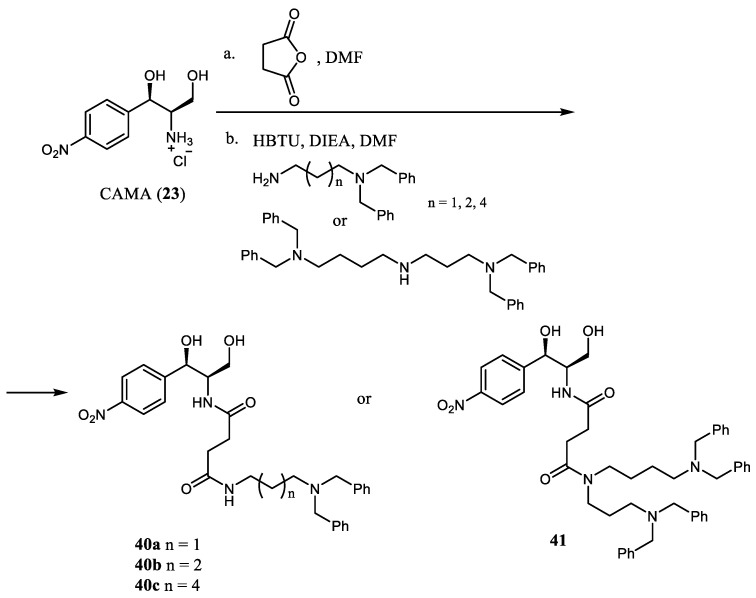
Synthesis of polyamine derivatives of chloramphenicol.

One-pot acylation of the chloramphenicol amine (**23**) with succinic anhydride followed by coupling with the N^1^,N^1^-dibenzylated linear diamines or N^1^,N^1^,N^8^,N^8^-tetrabenzylspermidine in the presence of HBTU and Et_3_N or DIEA in DMF resulted in chloramphenicol conjugates **40a**–**c** and **41**, respectively (Scheme 7) [20]. Previously, CAM conjugates with natural polyamines (PAs) have been described in an attempt to obtain less toxic phenicols [21,22]. Despite the fact that novel analogs **40a**–**c**, **41** were able to bind with the *E. coli* ribosome with affinities close to that of CAM (**1**) (K_i_ 0.8–2.6 µM vs. 1.5 ± 0.1 µM for **1**), they turned out to be inactive against Gram-positive and Gram-negative bacteria (MIC values against *E.coli* and *S.aureus* > 200 µM), presumably because of their low ability to penetrate the bacterial membrane. Nevertheless, compound **41** proved to be effective against human mesothelioma ZL34 and demonstrated moderate activity against immortalized mesotheliomal nontumor Met5A cells. The possible explanation of the antiproliferative activity of derivative **41** is related to its ability to penetrate ZL34 cells, using polyamine transporters present in cancer cell membranes and its inhibition action on the mitochondrial protein synthesis. This was demonstrated using cytochrome C oxidase subunit II (COX2) as a marker. Thus, the introduction of the polyamine substituents bears hydrophobic benzyl groups and can be considered an approach for finding promising anticancer agents with no adverse effects on bacterial microflora when taken orally.

### 2.3. α,β-Unsaturated Carbonyl Derivatives of CAM

**Figure 5:**
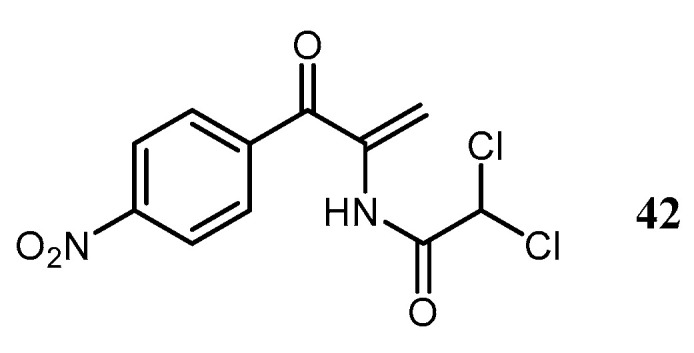
Structure of α-dichloroacetamido-*p*-nitroacrylophenone.

The α,β-unsaturated CAM analog, α-dichloroacetamido-*p*-nitroacrylophenone (Figure 5, **42**), was able to overcome resistance to CAM (**1**) in *St. aureus* which can be possibly explained by the lack of the primary and secondary hydroxyl groups that undergo acylation by acetyl- and phosphotransferases in resistant strains [23].

**Scheme 8:**
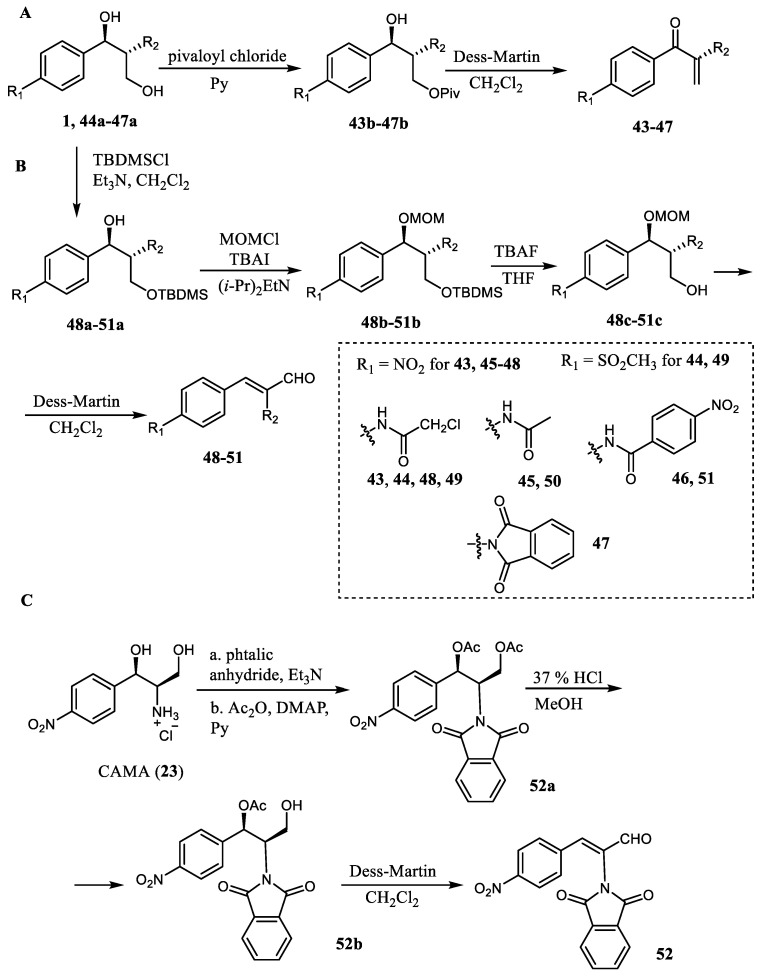
Synthesis of CAM-derived enone (route **A**) and enal analogs (routes **B**,**C**).

Zada et al. described the preparation and evaluation of the α,β-unsaturated carbonyl derivatives of chloramphenicol (**1**) and derivative **42** for SAR studies [24]. The target compounds were synthesized starting from commercially available CAM (**1**), thiophenicol (**44a**), or from the synthetic CAM analogs (**45a**–**47a**) (Scheme 8A). At the first stage, the primary hydroxyl group was selectively transformed to the pivaloyl ester, then the second group of the obtained compounds **43b**–**47b** was oxidized using Dess-Martin reagent. The chromatography purification of the intermediates was accompanied by the elimination of the *O*-pivaloyl group, resulting in the formation of the target enone compounds **43**–**47**. Enal derivatives **48**–**51** were prepared by a four-stage synthetic procedure from the same starting compounds (Scheme 8B). Selectively protected at the secondary hydroxyl group derivatives **48c**–**51c** were obtained after introduction of the TBDMS group at the primary hydroxyl group, the introduction of the MOM protecting group, and the selective deprotection of the primary hydroxyl. The primary hydroxyl group of **48c**–**51c** was oxidized using Dess-Martin reagent followed by flash chromatography on silica gel which was accompanied by the elimination reaction giving the enal derivatives **48**–**51**. Analog **52** was obtained starting from CAM amine (**23**) which was first transformed to the di-*O*-acetyl-N-phthalimide CAM derivative **52a**. The second stage included selective removal of the acetyl group from the primary hydroxyl group in the presence of HCl, resulting in the formation of the monoacetylated compound **52b**. The target enal derivative **52** was obtained after Dess-Martin oxidation followed by an elimination reaction during the flash chromatography purification.

The antibacterial activity of the synthesized compounds was evaluated on a wide panel of Gram-positive and Gram-negative bacterial strains and revealed that compounds **43** and **46** demonstrated MIC values 2–32 μg/mL against a wide panel of Gram-positive strains, including strains that are resistant to parent CAM (**1**) and thiophenicol. Obviously, the enone structural residue was essential for the antibacterial activity, as none of the compounds **48**–**52** have aldehyde function (enals). However, they did not exhibit antibacterial activity against the tested strains. Notably, derivatives **43** and **46** demonstrated a lower ability to induce the evolution of resistance in *S. aureus* than CAM (**1**) and were significantly less active translation inhibitors than the parent antibiotic (**1**). The short incubation of analogs **43** and **46** with Gram-positive cells caused the extensive deformation of bacterial cells, suggesting that they can have an effect on bacterial membrane integrity. In-depth studies of the effects of derivatives **43** and **46** demonstrated that, most likely, they inhibited the early stage of cell wall peptidoglycan biosynthesis in *S. aureus* cells, thus, indicating that these analogs have other modes of action than the parent CAM (**1**). Additional studies have confirmed that new CAM analogs did not possess hemolytic activity on rat red blood cells at concentrations 4- to 32-fold higher than their MIC values. However, unlike CAM (**1**), they exhibited cytotoxic activity against human lung carcinoma epithelial cells A540 and normal human bronchial epithelial cells BEAS-2B at the same range of concentrations as the MIC values.

Thus, the developed molecular scaffold on the basis of α,β-unsaturated carbonyl CAM derivatives can be useful for designing novel therapeutic agents since they act as inhibitors of bacterial cell wall biosynthesis. However, additional modifications of the structure are needed in order to reduce their toxicity towards human cells.

## 3. Discussion

Although CAM (**1**) has a broad spectrum of antibacterial activity, its clinical potential is seriously limited by serious side effects, such as hematologic disorders, immunosuppression, and cancer invasion due to the widely spread antimicrobial resistance to this antibiotic. This could be intrinsic or acquired by the use of this antibiotic as a veterinary drug and in clinics. Currently, CAM is prescribed in developed countries only in cases of serious infections, such as caused by *Haemophilus influenza,* or in patients with bacterial meningitis or brain abscesses who are allergic to other classes of antibiotics, such as penicillins. Numerous efforts to obtain chloramphenicol analogs with improved pharmacological properties have so far yielded two clinically useful CAM derivatives—thiophenicol and florphenicol, both having limited clinical potential and used mainly as veterinary drugs. As it is incredibly difficult to find CAM analogs with significantly improved pharmaceutical properties, the paradigm shift of investigating new CAM derivatives has occurred, and the most recent efforts for synthesizing new derivatives of chloramphenicol are concentrated more on elucidating the molecular basis of the mode of CAM action and the mechanisms of resistance. Most new chloramphenicol derivatives described since 2016 represent the amino acid or peptide conjugates of CAM used for detailed studies of CAM–ribosome interactions by molecular docking and biochemical methods. Other promising approaches are connected with the use of the CAM scaffold for discovering therapeutic agents with a mode of action that is different from chloramphenicol, such as selective antiproliferative activity or bacterial cell wall biosynthesis inhibition. Hopefully, a deeper understanding of the biology and the interactions of the drug, as well as a better understanding of CAM specificity, will generate new research ideas for developing novel, more effective drugs.

## Figures and Tables

**Table 1:** Antibacterial activity of compounds **1**–**7** and **10**–**16**.

Compound	IC_50_/MIC (μg/mL) ^1^	
	*M. intracellulare* ATCC 23068	*M. tuberculosis* ATCC 25177
CAM (**1**)	17.2 ± 0.02/25.0 ± 0	67.6 ± 16.7/− ^2^
**2**	18.9 ± 1.2/25.0 ± 0	−/−
**3**	32.3 ± 9.1/75.0 ± 35.30	−/−
**4**	12.6 ± 0.5/25.0 ± 0	−/−
**5**	−/−	−/−
**6**	23.8 ± 2.5/25.0 ± 0	−/−
**7**	−/−	−/−
**10**	20.2 ± 4.2/37.5 ± 17.7	29.0 ± 2.5/50.0 ± 0
**11**	45.3 ± 0.1/50.0 ± 0	−/−
**12**	40.7 ± 11.7/50.0 ± 0	57.0 ± 7.0/100.0 ± 0
**13**	15.4 ± 1.4/25.0 ± 0	−/−
**14**	32.2 ± 2.0/50.0 ± 0	−/−
**15**	27.5 ± 2.0/50.0 ± 0	87.7 ± 2.4/−
**16**	6.8 ± 0.2/12.5 ± 0	27.4 ± 6.2/50.0 ± 0

^1^ IC_50_—50% inhibition of bacterial growth; MIC—minimum inhibitory concentration (the lowest concentration that allows no detectable growth). ^2^ Not active at the highest concentration (100 μg/mL) used for the screening.

**Table 2:** Antibacterial activity of peptide-CAM conjugates **22** against *E. coli* K-12 (MIC, µM).

Compound	MIC, µM	Compound	MIC, µM
CAM (**1**)	20	**22q** CAMsu-GGGGG	40
CAMsu (**21**)	>200	**22aa** CAMsu-rr	200
**22a** CAMsu-GI ^1^	20	**22ab** CAMsu-kk	200
**22b** CAMsu-Ga	20	**22ac** CAMsu-KK	200
**22c** CAMsu-Gs	20	**22ad** CAMsu-ffrr	200
**22d** CAMsu-sG	40	**22ae** CAMsu-ffkk	200
**22e** CAMsu-GGNHMe	20	**22af** CAMsu-GK	20
**22f** CAMsu-GGGG	20	**22ag** CAMsu-ee	>200
**22g** CAMsu-aa	80	**22ah** CAMsu-dd	>200
**22h** CAMsu-AA	100	**22ai** CAMsu-DD	200
**22i** CAMsu-aaa	80	**22aj** CAMsu-GD	20
**22j** CAMsu-AAA	100	**22ba** CAMsu-ff	100
**22k** CAMsu-GGf	20	**22bb** CAMsu-ff(2-NaI)	>200
**22l** CAMsu-GGF	20	**22bc** CAMsu-ff(2-NaI)GG	>200
**22m** CAMsu-GFG	20	**22ca** CAMsu-a	>200
**22n** CAMsu-FGG	80	**22cb** CAMsu-OMe	>200
**22o** CAMsu-GGff	20	**22cc** CAMsu-NH(CH_2_)_2_SO_3_H	40
**22p** CAMsu-GGFF	20	**22cd** CAMsu-NH(CH_2_)_2_OPO_3_H	100

^1^ Standard 1-letter abbreviation for amino acids is used; in case of d-amino acid corresponding small (lowercase) letter was used.
